# Realistic genetic competencies achievable by US primary care providers

**DOI:** 10.1186/1472-6963-14-S2-P98

**Published:** 2014-07-07

**Authors:** Nadeem Qureshi, Kathleen Szegda, Joe Kai

**Affiliations:** 1 Division of Primary Care, University of Nottingham, Nottingham, UK; 2 Pioneer Valley Asthma Coalition, Springfield, USA

## Background

Genetic medicine is increasingly being incorporated into primary care predominately in preventive activity. Further, minority populations in the USA, as in other countries, are exposed to health disparities in many aspects of health service provision. Genetic medicine is in danger of amplifying these disparities. This could be counteracted by improving the genetic competency of Primary Care Providers (PCP). In the US, these providers include family doctors, general internists and paediatricians. Objectives of this qualitative study included:

(1) Map current genetic activity for minority populations within primary care/community setting in the US.

(2) Critically appraise skills, attitude and knowledge of primary care/community health professionals currently fulfilling a genetic role with underserved populations.

## Materials and methods

Documentary evidence was reviewed to inform the development of the interview schedule on primary care professionals’ competencies. Key informants were identified through contact with academic residency-training primary care practices and through US family practice (non-specialist) conferences. A series of 12 semi-structured interviews were completed with key informants. These interviews were transcribed and thematic content report back to informants to draw recommendations on future achievable competency criteria.

## Results

Certain attributes where identified that are required for PCPs to develop genetic competency. These included adapting communication skills, working with families and appreciating the local disease prevalence. These attributes are enhanced by PCP keeping up to date, using standard procedures, and presence of genetic literacy in consulting patients. However competency is hindered by lack of time and resources, poor awareness of patients’ beliefs, and inappropriate confidence in competency.

## Conclusion

PCPs’ genetic competency can be improved by incorporating transferable skills and concentrating on genetic conditions with higher prevalence in local area. This can be enhanced *by* improving the community’s genetic **literacy.**

